# How to treat gastrinomas in patients with multiple endocrine neoplasia type1: surgery or long-term proton pump inhibitors?

**DOI:** 10.1007/s00595-022-02627-z

**Published:** 2022-12-07

**Authors:** Masayuki Imamura, Izumi Komoto, Yoshiro Taki

**Affiliations:** 1 Neuroendocrine Tumor Center, Kansai Electric Power Hospital, 2-1-7, Fukushima, Fukushima-Ku, Osaka City, 553-0003 Japan; 2 Department of Surgery, Kyoto University Graduate School of Medicine, 54 Shogoin Kawaharacho, Sakyo-Ku, Kyoto City, 606-8507 Japan; 3 Department of Surgery, Kansai Electric Power Hospital, 2-1-7, Fukushima, Fukushima-Ku, Osaka City, Japan

**Keywords:** Gastrinomas, MEN1, Selective arterial secretagogue injection test (SASI test), Pancreas preserving total duodenectomy (PPTD), Proton pump inhibitors (PPIs)

## Abstract

In patients with multiple endocrine neoplasia type 1 syndrome (MEN 1) and Zollinger–Ellison syndrome (ZES), gastrinomas arise from the duodenum, about 60% are multiple, and about 15% of patients have coexisting pancreatic gastrinomas, which can be localized by the selective arterial secretagogue injection test (SASI test). The guidelines (GLs) by the Japanese Neuroendocrine Tumor Society (JNETS) recommend surgical resection for functioning duodenopancreatic neuroendocrine tumors (NETs), including gastrinomas, in patients with MEN1 (Grade A, 100% agreement among members). Conversely, the GLs of the National Comprehensive Cancer Network (NCCN) in the USA recommend observation and treatment with proton pump inhibitors (PPIs) or exploratory surgery for occult gastrinomas. An international Consensus Statement (ICS) from the European Union (EU) also does not recommend resection of gastrinomas in patients with MEN1, despite some surgeons having reported surgery being curative for gastrinomas in MEN1 patients. In this review, we discuss the serious side effects and tumorigenic effects of the prolonged use of PPIs and the safety and curability of surgery, supported by our results of curative surgery for gastrinomas in 20 patients with MEN1 over 30 years. We conclude that surgery should be the first-line treatment for gastrinomas in MEN1 patients.

## Introduction

In patients with multiple endocrine neoplasia type 1 syndrome (MEN1) and Zollinger–Ellison syndrome (ZES), gastrinomas usually arise from the submucosal layer of the duodenum. About 60% of these gastrinomas are multiple and about 15% of patients have pancreatic gastrinomas [1–6]. These gastrinomas have the potential to metastasize to the regional lymph nodes and liver, even if the primary tumors are less than 5 mm in diameter (Table 1) [1–16]. The guidelines (GLs) of the Japanese Neuroendocrine Tumor Society (JNETS) recommend resection for functioning duodenopancreatic neuroendocrine tumors (NET) in MEN1 patients (Grade A, 100% agreement among members) [6]. On the other hand, the National Comprehensive Cancer Network (NCCN) GLs in the USA do not recommend surgery initially for gastrinomas but advises either observation with treatment using proton pump inhibitors (PPIs) or exploratory surgery for occult gastrinomas [7]. An International Consensus Statement (ICS) from the EU also does not recommend resection of gastrinomas as an initial therapy for MEN1 patients [8].

**Table 1:** Characteristics of gastrinomas in patients with multiple endocrine neoplasia type 1(MEN1) and Zollinger–Ellison syndrome (ZES)

Location
All gastrinomas arise in the duodenum and about 60% are multiple
About 15% of patients have co-existent pancreatic gastrinomas [1–8].
Currently, the selective arterial secretagogue injection (SASI) test is the only useful technique to locate gastrinomas in MEN 1 patients. While ^68^Ga-DOTATOC (or DOTATATE)-PET/CT (SRI) can localize small neuroendocrine tumors (NETs), it cannot differentiate gastrinomas among multiple duodenopancreatic NETs in MEN1 patients [1, 4].
Malignant potential
The rate of nodal metastases of the duodenal gastrinomas is about 60%, even for gastrinomas less than 5 mm in diameter. It was reported that the rate of hepatic metastases reached 23–29% when the primary duodenal gastrinomas had not been resected, although it remained in the range of 3–5% when they had been resected [14–16].
Surgery aided by the results of the SASI test
1. When the gastroduodenal artery is identified as a feeder of gastrinomas and the splenic artery is not, we perform pancreas-preserving total duodenectomy (PPTD)
2. When the splenic artery is also identified as a feeder of gastrinomas by the stimulation via a small catheter placed at different points in the splenic artery, we perform local resection of the body or the tail of the pancreas to preserve the pancreatic endocrine function as much as possible
To treat coexisting nonfunctioning pancreatic NETs, pancreatic NETs more than 2 cm in diameter are indication for resection. A different kind of method of resection is performed according to the situation (enucleation, central resection of the pancreas, distal pancreatectomy or PD) [6].

Gastrinomas were first recognized in patients with severe complications from uncontrollable acid secretion secondary to hypergastrinemia [17]. Now, gastric acid hypersecretion can be suppressed by PPIs, thereby minimizing the risk of these complications [18, 19]. However, if MEN1 patients with gastrinomas are not treated by surgical resection, they must take PPIs for the rest of their life. For many patients, this may be 40 or 50 years and since hypergastrinemia persists for as long as the gastrin-producing tumors are present, PPI treatment must continue. Is this truly innocuous? Recently, serious side effects of the long-term use of PPIs have been reported, [20–25] as well as a tumorigenic effect of hypergastrinemia that continues with the prolonged use of PPIs [26–33].

Gastrinomas must be considered malignant since most eventually metastasize to the liver or intraperitoneal cavity [1–16]. This is not prevented by PPIs; therefore, it seems more logical to perform resection for the gastrinomas instead of using PPIs alone in patients. We discuss herein, the problems associated with the long-term use of PPIs, as well as the surgical technique we use for resection, and the morbidity and mortality of gastrinoma patients. We also report our results of performing curative surgery for gastrinomas in 20 patients with MEN1 over 30 years.

## Chronology of the treatment of gastrinomas in MEN1 patients

### History

**Fig. 1:**
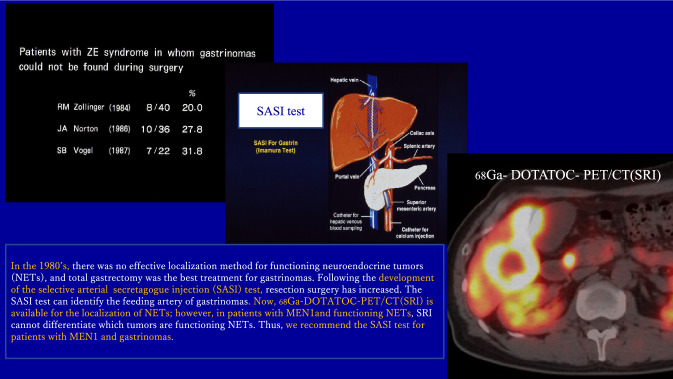
Patient with ZE syndrome in whom gastrinomas could not be found during surgery

In 1955, Zollinger RM and Ellison EH described two patients with severe recurrent peptic ulcers even after repeated ulcer operations including subtotal gastrectomy, thereafter named Zollinger–Ellison syndrome (ZES) [17]. Subsequently, surgeons tried in vain to cure gastrinomas, until the development of the selective arterial secretagogue injection (SASI) test [34]. In the 1980’s, the portal venous samplings were the only available technique to localize a functioning duodenopancreatic NET. This method would measure the hormonal levels in the portal system by drawing blood samples from several points through a catheter directly inserted into the portal vein [35]. However, this was invasive, and the results were unreliable. At that time, 20.0–31.8% of patients with ZES had gastrinomas that could not be found even by laparotomy (Fig. 1). [36–38] Zollinger recommended total gastrectomy as the best strategy to control the complications of unchecked acid secretion in patients with ZES. During this period, the patients died from their peptic ulcer complications [36]. In 1987, we developed a selective arterial secretin injection (SASI) test for the localization of gastrinomas and reported the results in four patients with ZES (Fig. 1) [34]. The principle of the SASI test is to localize gastrinomas or insulinomas by identifying a feeding artery of these functioning NETs; thus, it may be considered a physiological diagnostic method [34]. In 1989, we reported the results of curative resection of gastrinomas aided by the SASI test and the intraoperative secretin test of multiple occult duodenopancreatic gastrinomas for 11 patients with ZES, most of whom were MEN1 patients. In this series, 10 patients were biochemically cured. The one patient who was not cured had hepatic metastases [4, 13]. In 1990, Doppman et al. used the SASI test in 13 patients with ZES and reported that it was a useful method that should replace portal venous samplings [39]. Since the development of the SASI test, resection surgery for patients with gastrinomas has increased [4, 5, 13–16, 40]. Now we know that gastrinomas arise predominantly in the duodenum rather than in the pancreas in ZES patients with or without MEN1 [1–13]. There have been subsequent advances in both anti-acid medications and imaging techniques for NET.

### Now

PPIs became available in 1993 to prevent the complications of the hypersecretion of gastric acid in patients with gastrinomas [18, 19]. Somatostatin receptor imaging (SRI) with ^68^Ga-DOTA-conjugated somatostatin receptor targeting peptides can visualize NETs at least 5 mm in diameter (Fig. 1) [41]. Moreover, anti-NET medicines, including hormonal, chemical, or molecular targeting drugs, have been developed. Using these medicines, the growth of metastatic NETs can be partially suppressed, extending the survival of patients with metastatic NETs [6–8]. On the other hand, resection of gastrinomas in patients with MEN1 has not been recommended in the Western GLs [7, 8], although some surgeons in EU and Japan have performed curative surgery for gastrinomas in either sporadic or MEN1 patients using preoperative localization with the SASI test [1, 6, 10, 11]. Table 2 lists the characteristics of nonfunctioning pancreatic NETs and NETs arising in other organs in patients with MEN1 with the treatment strategies recommended by the JNETS GL.

**Table 2:** Treatment strategy for malignant and benign neoplasms in patients with MEN1 recommended by Japan Neuroendocrine Tumor Society (JNETS) Guidelines [6]

Pancreas
Multiple nonfunctioning micro- or macro-NETs exist diffusely throughout the whole pancreas. The malignant potential of these NETs increases with their size
Indications for resection of these NETs are a size of 2 cm or more in diameter and rapid growth. The JNETS guidelines recommend avoiding total pancreatectomy when possible, to preserve the pancreatic endocrine function
Thymus
NETs and neuroendocrine carcinoma (NECs) of the thymus are highly malignant
Curative resection with dissection of the regional lymph nodes of any size is recommended
Parathyroid
Hyperparathyroidism (HPT) caused by hyperplasia of the parathyroid glands occurs in more than 90% of MEN1 patients and results in hypercalcemia
Total parathyroidectomy with transplantation or subtotal parathyroidectomy is recommended. For patients with ZES, surgery for HPT is usually performed prior to surgery for gastrinomas
Pituitary
Most tumors are benign and slow growing
Observation or medical treatment is usually recommended, with resection generally only recommended for occasional symptomatic tumors

## Characteristics of gastrinomas in patients with MEN1 (Table 1)

Since the development of the SASI test, the number of gastrinomas from patients with MEN1 that have been resected and pathologically examined has increased. There is consensus that gastrinomas in MEN1 patients develop in the duodenum, about 60% are multiple, and about 15% of patients have co-existent pancreatic gastrinomas [1–16]. MEN1 patients have microgastrinomas and/or G cell hyperplasia developing in the duodenal Brunner glands [1, 3]. We consider these to be a cause of post-operative recurrence, so we recommend a total duodenectomy rather than an extirpation or a partial duodenectomy [1].

## What kind of surgery has been performed for gastrinomas in patients with MEN1?

Like us, pancreas surgeons in Europe have performed curative resections of gastrinomas guided by the SASI test and reported satisfactory results mainly after pancreatoduodenectomy (PD) in MEN1 patients [1, 5–16]. When the gastroduodenal artery is identified as the feeder of gastrinomas, and the splenic artery is not, surgeons have performed PD [8–13]. We also performed PD which clearly proved that gastrinomas in MEN1 patients are predominantly located in the duodenum [1–5, 9–13]. Now when the SASI test shows that the gastroduodenal artery and not the splenic artery is the feeder of gastrinomas, we principally perform pancreas-preserving total duodenectomy (PPTD) and enucleations of the tumors in the head of the pancreas [1, 42]. If the splenic artery is also a feeder, we perform enucleations or distal pancreatectomy in addition to PPTD [1, 42].

Dissection of lymph nodes (LN) around the head of the pancreas is very important, and recurrence from the para-aortic LN behind the head of the pancreas does occur. Because of this, we recommend dissection of the LNs along both the anterior and posterior arcade of the pancreaticoduodenal arteries and along the common hepatic artery, as well as the para-aortic LN just behind the head of the pancreas [1, 42]. We have performed enucleations or either a central- or a distal-pancreatectomy for nonfunctioning pancreatic NETs more than 2 cm in diameter [42].

In patients with MEN1, hyperparathyroidism often co-exists. For these patients, we have performed total parathyroidectomy with auto-transplantation or subtotal parathyroidectomy prior to the surgery for gastrinomas. This will typically cause a decrease in serum gastrin levels (Table 2) [1, 13, 34].

## Side effects of the long-term use of PPIs

If patients do not have a curative resection of gastrinomas, they must take high-dose PPIs for the rest of their life. Is this a safe strategy? The answer seems to be “No”. An increased number of articles have reported severe side effects of long-term PPIs use [20–26]. So, lifetime PPI use should not be considered innocuous. These reports showed that the prolonged use of PPIs can be accompanied by severe PPI-induced hypomagnesemia, resulting in paresthesia, seizures, muscle tremors, agitation, nausea, and/or cardiac arrythmias; calcium and vitamin B12 malabsorption; bone fracture; Clostridium difficile infection; community-acquired pneumonia; chronic kidney disease; and dementia [20–26].

Perrier et al. first reported a MEN1 patient with life-threatening hypomagnesemia, which became evident within 3 years of starting high-dose PPI therapy. [21] They do not believe in resection for cure of gastrinomas in MEN1 patients and did not perform an extirpation of the duodenal NET in this patient. To stop the need for PPIs, they performed total gastrectomy, an approach recommended in the 1980s before the development of the SASI test.

The true incidence of hypomagnesemia in patients on long-term PPIs is unclear, but the US Food and Drug Administration (FDA) has published an alert about the risks of hypomagnesemia resulting in serious complications [20–26]. According to Perrier et al. PPI-induced hypomagnesemia occurs after a median of 5.5 years of its use (range, 14 days to13 years) [21]. Risk of hypomagnesemia seems to increase with the concomitant use of loop diuretics and the duration of PPI therapy [21–26]. The main mechanism appears to be a pH-dependent regulation of the TRPM6 transporter in the colonic enterocytes, but an individual epigenetic polymorphism could explain why hypomagnesemia does not occur uniformly among PPI users [20–26]. A dose dependency of PPI-induced hypomagnesemia has not been described in the general population. Therefore, there are no guidelines regarding prevention and monitoring of drug-induced hypomagnesemia [20–26]. We believe that MEN1 patients with gastrinomas should be treated with PPIs in the short term to be better prepared for resection surgery.

## Anti-acid medicines cannot prevent the progression of gastrinomas, and the hypergastrinemia caused by both use of PPI and the gastrinomas may be tumorigenic

Gastric resection surgery for peptic ulcers has all but disappeared due to the development of H2 blockers and PPIs. [18, 19] Since PPIs can almost completely suppress the acid secretion in the stomach and prevent complications of hyperacidity such as the duodenal ulcer perforation, frequent diarrhea, and severe esophagitis [6–8], now we do not have to perform gastrectomy for patients with gastrinomas. However, PPIs cannot prevent the growth and progression of gastrinomas. It has also been reported that hypergastrinemia caused by both the secretion of gastrin from the gastrinomas and the resulting decrease in gastric acid production from the PPIs is tumorigenic and can accelerate the development of malignant tumors in the gastric mucosa [27, 28]. In 2004, Norton JA et al. reported five patients with MEN1 and ZES, who developed advanced-stage gastric carcinoid tumors during 21 ± 3 years observation. Total gastrectomy was performed in four patients, and hepatic metastases were found in three patients and lymph nodal metastases in four patients [28]. In 2016, Fossmark R et al. published [ECL-cell carcinoids and carcinoma in patients homozygous for an inactivating mutation of the gastric H ( +) K ( +) ATPase alpha subunit.] They found that homozygous siblings who were hypergastrinemic had gastric tumors diagnosed at a median age of 33 years. The tumors were NET G1 or G2. One of the NET G2 tumors also had a component of an intestinal type of adenocarcinoma [30]. In 2021, Ness-Jensen et al. reported that hypergastrinemia was associated with an increased risk of proximal gastric adenocarcinoma [27].

## Imaging techniques have not advanced to the level that can differentiate gastrinomas among multiple duodenopancreatic NETs (Fig. 1)

Today, ^68^Ga-DOTATOC (or DOTATATE)-PET/CT (SRI) is available and useful for the localization of NETs as small as 5 mm in diameter [41]. It is not unusual for patients with MEN1 to often have numbers of nonfunctioning NETs or even functional NETs such as glucagonoma, insulinoma, and VIPoma in the duodenopancreatic region. SRI, at present, cannot differentiate which NETs are gastrinomas or insulinomas among the many duodenopancreatic NETs in MEN1 patients. Thus, we recommended using the SASI test for the localization of functioning NET in MEN1 patients [1, 4, 13, 34]. (Fig. 1) Gastrinomas in MEN1 patients are typically located in the duodenum, whereas pancreatic gastrinomas co-exist in only a few patients [1–5]. If small submucosal tumors are found in the duodenum in MEN1 patients either endoscopically or with SRI, it is quite likely that these tumors are gastrinomas. This can be confirmed by endoscopic biopsy but determining if pancreatic gastrinomas co-exist or not requires performing the SASI test. We found that in MEN1 patients, microgastrinomas and/or G cell hyperplasia develop in the duodenal Brunner glands [1, 3]. These, we think, might be a cause of recurrence of duodenal gastrinomas, so we recommend total duodenectomy instead of local resection of the duodenal tumors [1].

## Results of our surgery for patients with MEN1 and gastrinomas

The study was performed under the approval by the Ethical Committee/IRB at Kansai Electric Power Hospital. (The approval number given by the Ethical Committee/IRB is 22-052). Our approach for gastrinomas in MEN1 patients without evidence of hepatic metastases for over 30 years has been to perform resection [1]. We investigated the outcomes and prognosis of 20 patients who have been followed up for more than 4 years postoperatively. Table 3 summarizes the results. All 20 patients had duodenal gastrinomas (100%), which were multiples in 12 (60%). Duodenal gastrinomas were 7 mm or less in diameter in 14 patients (70%) and were more than 10 mm in diameter in only two patients (10%). Pancreatic gastrinomas co-existed in three patients (15%). Lymph node metastases was detected in 13 patients (65%). Re-operation for recurrent duodenal gastrinomas was performed in two patients (10%).

**Table 3:** Results of curative surgery for gastrinomas in MEN type1 patients

#	Age y.o sex	Operation for gastrinoma	Location of gastrinoma (size: mm)		Mets. of gastrinoma		Nonfunctioning panc- NET size	HPT age at surg: y.o	Pit- NET age at surg: y.o	Prognosis, recurrence of gastrinoma
			D	P	N	L				
1	44 M	PD	1 (5)	0	1	0	5 1-2 mm	–	+ 41,42	No recurrence 18 years post-op, alive and well, then lost to follow-up
2	39 F	PD	7 (2–7)	0	1	0	6 1-2 mm	+ 39	–	No recurrence 4 years post-op, died of other disease
3	21 M	PD	1 (10)	0	0	0	0	–	+ 21	No recurrence 25 years post-op, alive and well
4	49 M	DX, DP	9 (1–7)	0	0	0	1 15 mm (gluc)	+ 47	+ 59	No recurrence 25 years post-op, alive and well
5	61 F	DX (twice), P-Enucl	4 (1–4)	2	1	0	2 3 mm, 5 mm	+ 55	–	No recurrence 14 years post-op, alive and well, then lost to follow-up
6	56 F	DX(twice) P-Enucl	2 (1–3)	0	0	0	3 3-10 mm	+ 55	–	No recurrence 21 years post-op, alive and well
7	44 F	DX	1 (9)	0	0	0	1 (PD 36 yo) 8 cm	+ 44	–	No recurrence 21 years post-op, alive and well
8	33 M	DX, DP	1 (10)	0	1	0	Multiple gluc	+ 23, 33	+ 50	No recurrence 19 years post-op, alive with post-craniotomy complications
9	54 F	DX,	2 (6, 12)	1 15 mm	3	0	Multiple insulinomas (DP:23 y)	+ 34	+ Γknife 23, 40	No recurrence 12 years post-op, alive and well
10	59 F	DX	1 (7)	0	1	0	0	+ 59	+	No recurrence 12 years post-op, alive and well
11	51 F	PPTD	Multi (1–4)	0	2	+ –	9	+ 51	–	Died of liver mets. 6 years post-op, probably as a result of non-curative surgery due to liver metastases
12	30 M	PPTD, DP	1 (5)	0	0	0	2	+ 30	+	18 years post-op, alive with liver mets
13	32 M	PPTD	2 (5, 7)	0	1	0	Multi 1-5 mm	+ 31	+	No recurrence 18 years post-op, alive and well
14	48 F	PPTD	Multi (1–5)	0	1	0	Multi 2-9 mm	+ 48	–	15 years post-op, alive with liver mets controlled by somatostatin analog
15	33 M	PPTD	1 (8)	0	2	0	Multi 1-5 mm	+ 26	–	14 years post-op, alive with liver mets. after resection of NETG2 of thymus (49 yo)
16	57 F	PPTD	7 (< 2)	0	1	0	0	+ 56	–	No recurrence 5 years post-op, died of other disease
17	32 M	PPTD Enucl, DP	12 (2–4)	2	3	0	Multi < 5 mm	+ 32	–	12 years post-op, alive and well but with #16 LN met
18	68 M	PPTD	8 (2–13)	0	3	0	3 (gluc)	+ 68	+	No recurrence 10 years post-op, alive and well
19	62 M	PPTD	2 (3–5)	0	0	0	Multi < 10 mm	+ 42	+	No recurrence 8 years post-op, alive and well
20	47 M	PPTD	1 (7)	0	0	0	Multi < 13 mm	+ 47	–	No recurrence, 4 years post-op, alive and well

Details of the patients with multiple endocrine neoplasia type 1 (MEN1) who underwent resection surgery for gastrinomas by our team

All 20 patients (100%) had duodenal gastrinomas, which were multiple in 12 (60%). Duodenal gastrinomas were 7 mm or less in diameter in 14 patients (70%) and more than 10 mm in diameter in 2 patients (10%). Pancreatic gastrinomas co-existed in 3 patients (15%). Lymph node metastases were detected in 13 patients (65%). Re-operation for recurrent duodenal gastrinomas was performed in 2 patients (10%). Biochemical cure of the gastrinomas was achieved in 15 patients (75%), who have remained well without evidence of recurrence of gastrinomas for 15.0±7.1 years (4–25 years). Two patients (10%) died of other diseases, 4 and 5 years postoperatively, respectively. Hepatic metastases developed postoperatively in four patients (20%). One of them died of progression of the gastrinomas 6 years postoperatively, and the other three have remained well for 14, 15, and 18 years respectively, on anti-NET medicines. Lymph node metastasis developed postoperatively in one patient (5%) who has remained well on anti-NET medicines for 12 years. For distant metastases that developed postoperatively, we gave somatostatin analogs, chemotherapy, molecular targeting agent or peptide receptor radionucleotide therapy (PRRT)

*M* male, *F* female, *Panc-* pancreatic, *y.o.* years old, *HPT* hyperparathyroidism, *NET* neuroendocrine tumors, *PD* pancreatoduodenectomy, *DX* partial duodenal resection, *D* duodenum, *P* pancreas, *N* lymph node metastasis, *L* hepatic metastasis, *mets* metastases, *post-OP* postoperatively, *P-enucl* enucleation of a pancreatic neuroendocrine tumor, *multi* multiple, *DP* distal pancreatectomy, *γknif* gamma-knife therapy, *PPTD* pancreas-preserving total duodenectomy, *gluc* glucagonoma, + positive, − negative, *#* No.

Biochemical cure of gastrinomas was achieved in 15 patients (75%), who have been well without evidence of recurrence of gastrinomas for 15.0 ± 7.1 years (4–25 years). Two patients (10%) died of other diseases 4 and 5 years postoperatively, respectively. Hepatic metastases developed postoperatively in four patients (20%), one of whom died of gastrinomas progression 6 years postoperatively while the other three have been alive and well for 14, 15, and 18 years, respectively, with the use of anti-NET medicines. Lymph node metastasis developed postoperatively in one patient (5%) who has been alive and well on anti-NET medicines for 12 years. For distant metastases that developed postoperatively, we have used somatostatin analogs, chemotherapy, a molecular targeting agent, or peptide receptor radionucleotide therapy (PRRT).

**Fig. 2:**
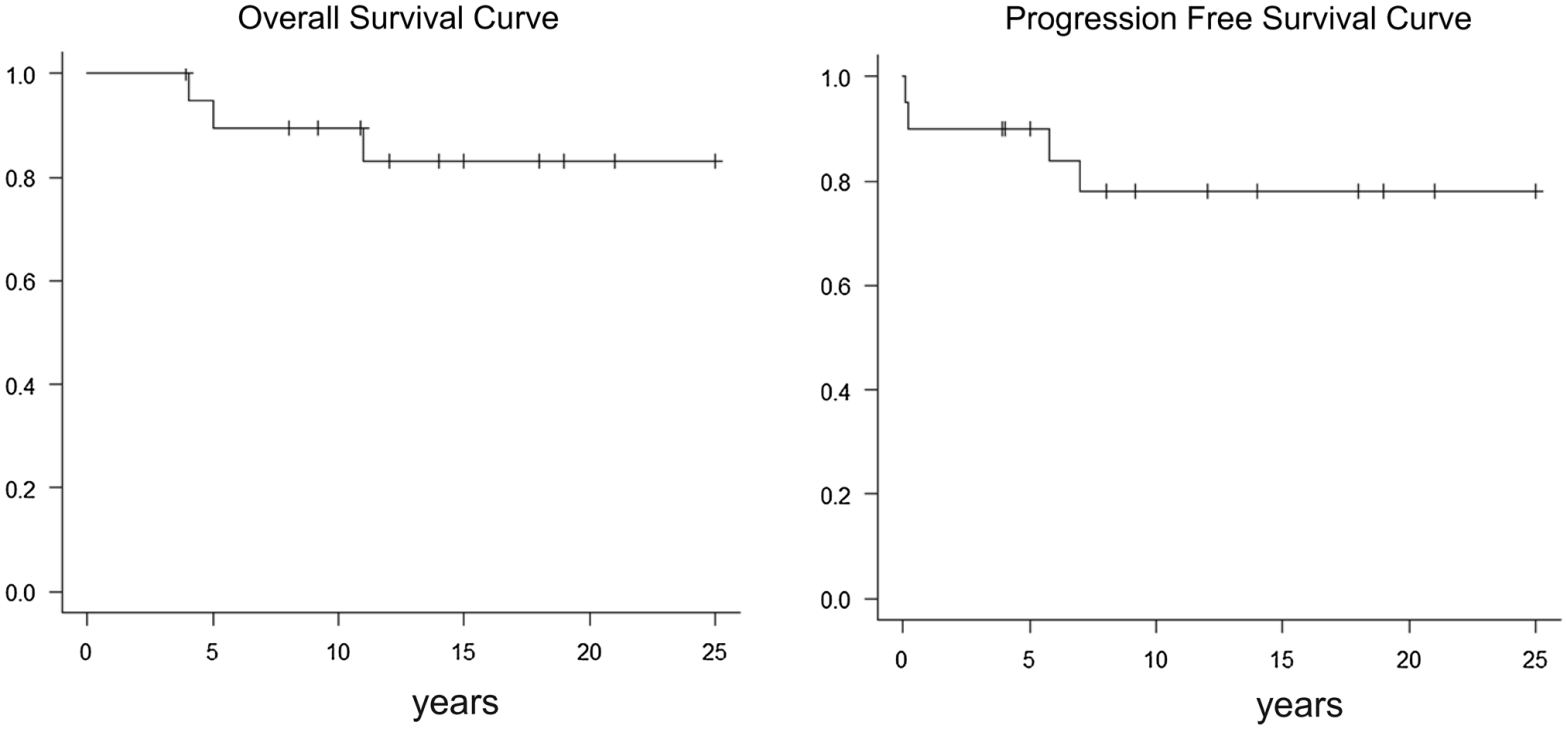
Overall survival (OS) and progression-free survival (PFS) of 20 patients with MEN1/gastrinomas who underwent surgery for gastrinomas in our center

Figure 2 shows the overall-survival (OS) curve and the progression-free survival (PFS) curve of these 20 patients. The OS rate and PFS rate at 25 years postoperatively were 80% and 78%, respectively.

## Discussion

Our surgical results for gastrinomas in MEN1patients highlight a few key facts about the characteristics of gastrinomas in MEN1 patients. First, gastrinomas grow in the duodenum in MEN1 patients with ZES, while pancreatic gastrinomas co-exist in only 15% of patients. Second, lymph node metastases were detected in 65% of patients at surgery, and hepatic metastases developed postoperatively in 20% of patients. It is described in the ICS from the EU, “At least 70–80% of MEN1 gastrinomas have been demonstrated to be malignant at the time of diagnosis and to show lymph node and/or liver metastasis, although the primary D-NEN(s) may be microgastrinomas as small as 1–2 mm in size (LE: 2b)” [6 articles are cited] [8]. They are of the opinion that most MEN1 gastrinomas are too malignant to be curable even by resection surgery. They cited six articles. We reviewed these articles, and found that in one, the authors described successful curative resection with PD for gastrinomas in MEN1 patients [9]. We have been able to perform biochemically curative surgery for gastrinomas in 75% of MEN1 patients. Thus, we do not think this description is correct and that curative resection can be performed if you will intend it. Following this sentence, they continue “Recent expert guidelines have suggested medical management using PPIs for most patients since the course of disease is rather mild, even without surgery. Based on the published literature, MEN1–ZES is considered a surgically non-curable disease (LE: 3b)”. This statement is based upon a single review article written by a medical researcher of MEN1 [44]. Recently, Kong W, et al. described that “some experts do not consider MEN1–ZES a surgically curable disease and therefore advocate a non-surgical approach using proton pump inhibitors (PPIs) to control the symptoms of hypergastrinemia [11]. It has been shown that 90% of patients with MEN1–ZES without pancreatic tumors on imaging are alive 15 years after the diagnosis, and even with diffuse distant metastases, the 10-year survival rate is around 54%. Considering the young average age of diagnosis of MEN1–ZES (around 40 years), one might argue that an overall survival of 15–20 years results in a substantial number of patients with MEN1 and gastrinomas dying before the age of 60. This is not a reasonable goal in modern healthcare” [11].

Before concluding that MEN1–ZES is not a surgically curable disease, the experts should have examined the results of curative surgery for these patients. The perioperative mortality rate was 0% in the report from Kong W et al. although 20% required reoperation for operative complications [11]. As shown in Table 3, we have been able to perform curative surgery for gastrinomas in 75% of MEN1 patients without a post-operative death. No reoperation was needed for operative complications. Thirteen of our 20 patients are well beyond the age of 60 (seven patients are 60–69 years old and six patients are 70–79 years old) (Table 3). Medical treatment for gastrinomas with long-term PPIs may result in reduced life expectancy of less than 60 years; risks of severe side effects like hypomagnesemia, which is the subject of an FDA alert [20–26]; and tumorigenic effects on the gastric mucosa. Conversely, surgical treatment does offer the only potential for cure as achieved in 75% of our MEN1 patients with gastrinomas without any post-operative mortality. The post-operative survival curves of our patients show OS and PFS rates after 25 years of 80% and 78%, respectively. Surgical treatment has made possible survival beyond the age of 80, which is a reasonable goal in modern healthcare.

Considering these facts, we think that surgical treatment should be the initial treatment recommendation for gastrinomas in MEN1 patients, instead of long-term PPIs. Some surgeons may think that PPTD is a difficult procedure, but it has been accepted internationally as less invasive for duodenal tumors because it does not include resection of the pancreatic parenchyma [42–44]. According to Cantalejo-Diaz M et al. who systematically reviewed reports of 211 patients who underwent PPTD for various diseases, including familial adenomatous polyposis and neuroendocrine tumors, the mortality rate was less than 1.4% [45]. In our series of PPTD for 10 patients with MEN1 and gastrinomas, the mortality rate was 0% and the morbidity rate was less than 10%. Postoperative development of diabetes mellitus has not been observed in our patients. If the pancreas divisum with an unfused pancreatic duct system is encountered, the accessory pancreatic duct must not be tied. It must be anastomosed to the jejunum; otherwise, acute pancreatitis will develop. [42] Our surgical results should encourage pancreatic surgeons to perform curative surgery for gastrinomas in MEN1 patients.

## Conclusion

Curative surgery should be recommended for gastrinomas in patients with MEN1.
